# Recent advances in understanding and preventing oesophageal cancer

**DOI:** 10.12688/f1000research.21971.1

**Published:** 2020-04-21

**Authors:** James Franklin, Janusz Jankowski

**Affiliations:** 1 Gastroenterology and Endoscopy Department, Kings Mill Hospital NHS Foundation Trust, Sutton-in-Ashfield, Nottinghamshire, NG17 4JL, UK; 2 University of Liverpool, Liverpool, UK; 3 University of Roehampton, London, UK

**Keywords:** oesophageal cancer, Barrett’s oesophagus, screening, surveillance

## Abstract

Oesophageal cancer is a common cancer that continues to have a poor survival. This is largely in part due to its late diagnosis and early metastatic spread. Currently, screening is limited to patients with multiple risk factors via a relatively invasive technique. However, there is a large proportion of patients diagnosed with oesophageal cancer who have not been screened. This has warranted the development of new screening techniques that could be implemented more widely and lead to earlier identification and subsequently improvements in survival rates. This article also explores progress in the surveillance of Barrett’s oesophagus, a pre-malignant condition for the development of oesophageal adenocarcinoma. In recent years, advances in early endoscopic intervention have meant that more patients are considered at an earlier stage for potentially curative treatment.

## Introduction

Worldwide, oesophageal cancer is the sixth most common cause of cancer-associated death, with 508,585 deaths reported in 2018 ^1^. Since the early 1990s, incidence rates have increased; however, over the past decade, the incidence rate has stabilised, with currently 9,100 new cases reported in the UK every year, equating to 25 every day ^2^. The prognosis remains poor, as diagnosis is often late owing to a lack of early easily identifiable symptoms. The survival rate has tripled over the past 40 years; however, the 10-year survival still remains poor at only 12% ^2^.

Oesophageal cancer can be subdivided into two main histological types: adenocarcinoma (OAC), normally affecting the lower third of the oesophagus and often preceded by Barrett’s oesophagus (BO), a recognised pre-malignant condition, and squamous cell carcinoma (OSCC), affecting the upper two-thirds of the oesophagus ^3^. The risk factors for OSCC include smoking and alcohol consumption, whereas for OAC, obesity, Caucasian race, gastro-oesophageal reflux disease (GORD), smoking, poor high-fat diet, and family history are widely recognised risk factors ^4^. Historically, OSCC has been the most prevalent subtype; however, particularly in developed countries such as the UK, the USA, and Australia, it has now been superseded by OAC ^5^. This is likely a result of the increase in GORD caused by obesity and poor diet, which directly contributes to the development of BO. The relative risk of OAC in BO patients was found to be 11.3 ^6^; this is comparable to the risk of breast cancer in first-degree relatives with a BRCA1/BRCA2 mutation with breast cancer ^7^.

**Figure 1.**
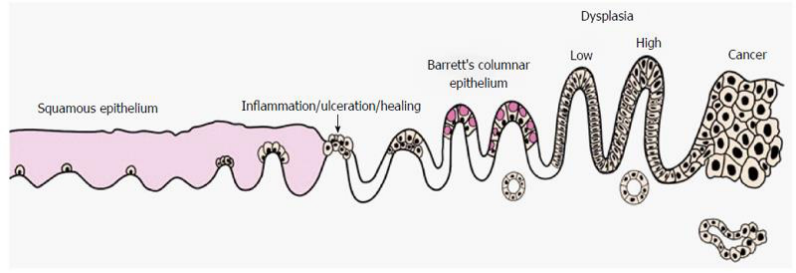
Development and progression of Barrett’s oesophagus to oesophageal adenocarcinoma. Re-printed from Evans *et al*., 2016 ^16^ licenced under CC BY-NC 4.0.

BO is the metaplastic change of the normal squamous oesophageal epithelium to columnar epithelium (see Figure 1). The severity of BO is graded on the presence and degree of dysplasia assessed via histopathological analysis of biopsy samples. The annual cancer risk for non-dysplastic BO is estimated at 0.33%, for low-grade dysplasia (LGD) 0.54%, and for high-grade dysplasia (HGD) 7% ^8^.

## Chemoprevention

Proton pump inhibitors (PPIs) are routinely prescribed in patients with BO with a view to reduce acid reflux, a key component in its pathogenesis. A systematic review of observational data showed that a reduction in acid suppression, through the use of PPIs, can reduce the risk of neoplasia ^9^. More recently, the chemopreventive properties of aspirin in OAC have also been explored. AspECT, a randomised prospective study, has evaluated the efficacy and safety of aspirin (300–325 mg) with a PPI (esomeprazole 40 mg twice daily or 20 mg daily) in BO patients. High-dose PPIs in combination with aspirin were found to be most beneficial to patient outcomes, including reducing mortality, OAC, and HGD ^10^. Although concerns have been raised regarding the safety of prescribing aspirin, which has known serious side effects, including major haemorrhage and death, in light of the aggressive nature of OAC, coupled with the often-late presentation and poor survival rates, there is an argument for a strong benefit:risk ratio. The current British Society of Gastroenterology (BSG) and American College of Gastroenterology (ACG) guidelines do not promote the routine use of aspirin in BO; however, further population-based studies are warranted to explore the potential long-term chemopreventive implications of aspirin.

## Screening

Currently, all national and worldwide screening guidelines recommend against general population screening and instead recommend limiting screening to patients with associated risk factors such as male gender, chronic GORD symptoms, >50 years of age, Caucasian ethnicity, obesity, smoking, and family history of BO/OAC. However, the number and severity of these risk factors differ substantially between organisations; as a result, there are variations in the screening of patients depending on where they live, even within a single country. Often those diagnosed with OAC do not have a prior diagnosis of BO ^11^, and more than a third deny a history of symptomatic reflux disease ^12^. This suggests that there is a need for a larger-scale screening program that is not limited to individuals presenting with GORD symptoms. There is evidence that screening for OAC leads to earlier detection and better outcomes, with the 5-year survival rate increasing from 17 to 74% ^13^.

The current gold standard for BO screening is oesophago-gastro-duodenoscopy (OGD) with associated biopsies following the Seattle protocol ^8, 14, 15^. An OGD is a relatively invasive and expensive procedure that is not well tolerated by some patients and is associated with a small risk of complications such as bleeding and perforation, making it an unideal tool for large-scale population-based screening ^17, 18^. For wider-scale screening to be implemented, there is a need for the development of novel strategies capable of early diagnosis of BO and OAC that are minimally invasive and affordable without increasing the burden on endoscopic surveillance programs. Several screening strategies are currently in development.

### Unsedated transnasal endoscopy

Unsedated transnasal endoscopy (uTNE) is an endoscopic technique that involves inserting an ultrathin endoscope transnasally with the use of topical anaesthetic. It is well tolerated in patients and has an excellent safety profile ^19, 20^. It has been shown to provide an assessment of the oesophagus of a quality comparable to that of OGD, with a sensitivity and specificity for BO of 98% and 100%, respectively ^21^. uTNE can easily be performed in a non-hospital setting, allowing novel mobile screening at a significantly lower cost ^22^. Further benefits of uTNA are a shorter evaluation time and quicker patient recovery of 15 minutes versus 1 hour ^23, 24^.

### Cytosponge ^TM^

Cytosponge ^TM^ is a novel transoral non-endoscopic cell collection device. It comprises a gelatine capsule on a string that is swallowed into the stomach, where it expands to 3 cm in 5–10 minutes. As it is retrieved, it collects cells from the oesophagus lining before being cytologically analysed for the biomarker Trefoil Factor 3 (TFF3), a key component of BO identification ^25^. The largest study to date to investigate the efficacy of the Cytosponge ^TM^ is the BEST2 study, which found a sensitivity of 80% that increased to 87% in patients with ≥3 cm of BO and a specificity of 92% ^26^. It was rated more favourably than OGD by patients, with a high success rate of 91–94% of patients able to swallow the device, most on the first attempt ^26, 27^. Cost effectiveness models have estimated a reduction in screening costs of 38–41% compared to endoscopic screening in patients with GORD symptoms ^28^.

### Balloon cytology

Balloon cytology is another non-endoscopic technique capable of obtaining a DNA sample from the distal oesophagus using a specifically designed textured balloon followed by molecular cytology analysis ^29^. Two molecular diagnostic markers for BO (cytosine methylation of *CCNA1* and *VIM* DNA) have been identified and have been reported to detect BO with a sensitivity and specificity greater than 90% ^29, 30^. The device is well tolerated, with 82% able to swallow the device and little or no reported anxiety or pain ^29^. Furthermore, 95% would recommend the procedure to others.

### Oesophageal capsule endoscopy

Oesophageal capsule endoscopy (OCE) is a non-invasive microscopic imaging technique requiring the use of a tethered camera encased in a vitamin-sized capsule that is swallowed transorally ^31^. It is already commercially available and is widely used for the investigation and diagnosis of various lower gastrointestinal conditions ^32^. It is only recently that its potential use for BO screening has been explored. A pooled sensitivity of 77% and pooled specificity of 86% have been reported by a meta-analysis of nine studies (n = 618) ^33^. It is safe and well tolerated with a high patient preference score, with 80% preferring OCE to OGD ^34^. However, it has not been found to be more cost effective, and there are concerns over its validity due to lack of biopsy samples with this technique ^35^.

### Liquid biopsy

Liquid biopsy involves the identification of DNA-encoded markers that are uniquely expressed or dysregulated in BO via a blood sample. A systematic review of 11 studies identified several microRNAs as promising biomarkers for diagnosing BO ^36^. A recent study reported a sensitivity of 78% and specificity of 86% when identifying four circulating microRNAs ^37^. This technique is in its relative infancy, and further refinement of relevant biomarkers is required before there can be a recommendation for its clinical use.

### Exhaled volatile organic compounds

A new technology that is not commonly used in diagnostic medicine is the detection of subtle changes in volatile organic compounds (VOCs) that can occur in altered disease states and can be detected by electronic nose (e-nose) devices from exhaled breath. Distinctive exhaled VOC profiles have been identified that are capable of distinguishing BO from controls ^38^. In a recent study comparing 66 BO patients to 56 controls, the e-nose was able to detect BO with a sensitivity of 82% and a specificity of 80% ^39^. This is an attractive method of screening, as it is non-invasive, potentially cost effective, and easily implemented in a primary care setting. Further studies are required to validate its use on a larger scale as well as its development for possible identification of dysplasia grade from VOC levels.

## Surveillance

BO is classified as the pre-malignant stage of OAC, and the recommended current method of surveillance for patients with diagnosed BO is white light endoscopic surveillance accompanied by quadratic biopsies at 2 cm intervals. The interval of surveillance is dependent on the length of metaplasia because of a positive correlation between length and OAC risk. The arbitrary cut off of 3 cm is considered higher risk and therefore a shorter surveillance period is recommended ^15^.

The aim of surveillance is to detect cancer at an early, potentially curable stage, crucially before the invasion of the submucosa, which increases the risk of lymph node metastasis ^40^. There is debate regarding the benefit of surveillance given the financial and resource burden it brings to gastroenterology departments along with potential complication risks, which many feel do not outweigh its long-term efficacy given the low risk of progression. However, a recent large-scale cohort study (n = 30,000) found that patients diagnosed with OAC during surveillance were detected at an earlier stage, had improved survival, and had a lower cancer-related mortality compared to those diagnosed in a non-surveillance program ^41^. The eagerly awaited results of the BOSS trial, a RCT comparing two-yearly endoscopic surveillance versus an ‘at need’ endoscopy, aims to shed light on the efficacy of this surveillance method and also its cost effectiveness ^42^. This surveillance strategy is time consuming and expensive, and the development of various image enhancement techniques has been proposed to improve its accuracy, reliability, and diagnostic capability. The implementation of these techniques could be potentially revolutionary, with one of the shortcomings of the current strategy being the difficulty in the histopathological analysis of dysplasia, which currently requires the input of two GI pathologists. Although these advanced imaging techniques are not currently commercially available or recommended in clinical guidelines, it has been estimated that they could increase the diagnostic yield for detection of dysplasia and OAC by 35% ^43^. Furthermore, recent promising work in the development of a p53 immunostain could further enhance diagnostic reproducibility and play a role in prognosis prediction ^44^.

### Dye-based chromoendoscopy

In order to overcome the limitations of white light endoscopy, dye-based chromoendoscopy was developed utilising a dye for mucosal and image enhancement during endoscopy. The dyes investigated include methylene blue, indigo carmine, and acetic acid. The most compelling evidence has been shown with the use of acetic acid, with a recent meta-analysis reporting a sensitivity of 92% and a specificity of 96% for HGD and OAC ^45^. Although this technique is cheap and universally available, a lack of RCT data has halted its implementation in routine clinical practice.

### Optical/digital chromoendoscopy

Optical/digital chromoendoscopy utilises narrow band imaging (NBI), a well-recognised advance in endoscopic imaging. In contrast to white light imaging, optical filters are used to narrow the wavelength to blue and green, enhancing the visualisation of microvasculature ^46^. It has been reported as highly accurate, with a higher sensitivity and equivalent specificity compared to traditional white light endoscopy ^47^, as well as requiring a reduced number of biopsies ^48^. This is a simple technique that can be easily implemented worldwide in combination with current white light endoscopy surveillance.

### Virtual chromoendoscopy

Virtual chromoendoscopy is based on the principle of digital post-processing such that endoscopic images are reconstructed, in real time, into virtual images without the need for dyes. Different filters and image algorithms can be applied to these images to further improve the visualisation and enhancement of the superficial structures of the mucosa and the mucosal microvasculature. The use of virtual chromoendoscopy has been shown to improve dysplasia detection sensitivity from 69 to 78% as well as improve specificity from 70 to 81% compared to white light endoscopy ^49^. This improvement was shown in both a trainee and an expert group of endoscopists and could result in a reduction in the need and number of biopsies required or greater targeting of biopsies.

### Confocal laser endomicroscopy

Confocal laser endomicroscopy (CLE) allows *in vivo* histological assessment with 1,000x magnification using a blue laser light following intravenous fluorescein administration. The high-resolution images produced are comparable to histological evaluation. There is a great future potential for this technique as a diagnostic tool, allowing interventional steps such as resection or ablation to be performed at the point of care ^50^. CLE has been shown to have a high sensitivity (90.4%) and specificity (92.7%) and reduces the number of biopsies taken, with one study reporting that two-thirds of patients did not require biopsies at all ^51^. Despite this, the technique is considered relatively expensive and lacks standardised criteria for diagnosis ^52^.

### Volumetric laser endomicroscopy

Volumetric laser endomicroscopy (VLE) is an imaging technique that scans the oesophageal surface in 360 ^o^ to a depth of up to 3 mm ^53^. It has the capability of visualising mucosal lesions invisible to white light and is expected to facilitate targeted biopsies ^54^. It has been found to be safe and accurate, with an adequate sensitivity (86%) and specificity (88%) ^55^.

### Artificial intelligence

Artificial intelligence (AI) is a brand new and promising research area across medicine as a whole. The development of AI for BO identification has been largely focussed on the identification of BO and OAC from endoscopic images and videos through auto-analysis ^56^. For example, the analysis of VLE images is complex, with approximately 1,200 cross-sectional images produced for each 6 cm oesophageal segment scanned, so the development of image analysis software could be considerably beneficial for the endoscopist ^57, 58^. Further validation is needed to ascertain its accuracy; however, AI has a potential role in aiding the surveillance of BO.

## Early endoscopic intervention

Historically, regular surveillance and in some cases radical oesophagectomy have been the only options available for the treatment of BO. Many patients have been reluctant to undertake such a major operation for a pre-malignant condition, and BO patients are often older and overweight with associated cardiac and respiratory co-morbidities, making them unfit for such an operation. Over the past decade, significant advances in endoscopic techniques have been developed that have broadened the options available to patients with both HGD and LGD for early intervention strategies.

Crucially, these techniques are performed endoscopically rather than surgically, which carries a high mortality rate ^59^. This includes developments in ablation and resection-based strategies, which have been shown to have a high level of efficacy. The less-invasive nature of these techniques has meant that more patients can be considered at an earlier stage in diagnosis for potentially curative treatment.

### Ablation techniques

Current guidelines from the ACG and BSG agree that ablation techniques such as radiofrequency ablation (RFA) and photodynamic therapies (PDTs) are promising emerging techniques in BO treatment. Both techniques aim to ablate anomalous oesophageal mucosa, causing tissue necrosis and regrowth of neo-squamous mucosa.

RFA uses controlled pulses of radiofrequency waves to thermally ablate the affected area. A RCT comparison of RFA against a control (sham procedure) (n = 127) reported complete eradication of dysplasia of 91% in LGD and 81% in HGD compared to 23% and 19%, respectively, in the sham procedure. Furthermore, eradication of metaplasia was 81% and 74% in LGD and HGD compared to 4% and 0%, respectively, in the sham procedure ^60^. Even more importantly, a RCT comparison of RFA against surveillance in LGD patients also found a significant reduction in dysplasia and metaplasia as well as a reduced risk of progression to OAC ^61^. The main complication with RFA was found to be strictures in 8–12% of patients, all of which were able to be successfully treated with endoscopic dilatation ^61, 62^.

PDT is a technique that involves the use of a photosensitising agent injected intravenously, which increases the sensitivity of the oesophagus lining to light. This agent is activated with a red-light laser, resulting in destruction of the abnormal epithelial cells through a photochemical as opposed to a thermal reaction as in RFA ^63^. The rate of dysplasia eradication with PDT in HGD has been found to range from 40–77% ^15^. Complications following PDT were high, with oesophageal strictures reported in 23–34% of patients ^64, 65^, skin reactions in a third ^66^, and oesophageal perforation, pleural effusion, and atrial fibrillation in 3–4% ^67^.

RFA has been shown to achieve a high rate of eradication of metaplasia and dysplasia as well as a reduction in disease progression ^68^. The safety and efficacy of RFA are well documented, and it is currently the most commonly used ablation technique ^69^. Current NICE guidance advocates the use of RFA for LGD and HGD and PDT for HGD ^70^. PDT has a significant photosensitivity and stricture risk; therefore, although good outcomes have been reported, it has largely been already replaced by RFA. Other ablation techniques are in development, such as cryotherapy ^71^; however, there is a lack of evidence for its safe use and efficacy, with a lack of RCTs and long-term follow up data.

### Resection-based strategies

Endoscopic mucosal resection (EMR) was first developed in 1978 for gastric cancers ^72^ but has since been adapted for BO treatment. Two common methods of EMR have been developed: the cap and snare technique and band ligation. In the cap and snare technique, the lesion is marked for resection using electrocautery and then injected with fluid. A dedicated transparent cap on the distal end of the endoscope is used. A preloaded crescent-shaped snare forms a pseudopolyp through suction of the lesion, which is then removed with a diathermy loop ^73^. This method has been reported to have a 91% neoplasia and metaplasia eradication rate at 18-month follow up but a high stenosis rate of 40% ^74^. The band ligation method creates a pseudopolyp using a band to ligate the lesion. It is then removed similarly to the cap and snare technique with diathermy ^73^. The band ligation method has been shown to be faster and cheaper than the cap and snare method as well as having a 92.3% complete resection rate ^75, 76^. The complication rate was also lower, with a stenosis rate of 1.9% ^77^. A systematic review comparing EMR and RFA reported equal efficacy in the treatment of BO; however, EMR encountered a higher proportion of complications ^78^.

An alternative to EMR is endoscopic submucosal dissection (ESD), which is a longer and more-advanced procedure that resects the lesion and deeper tissues, allowing a potentially larger clearance margin. Owing to the complexity of the procedure, it is currently only available in specialist centres.

The added benefit of resection-based strategies is that the tissue sample removed can be assessed; this has been found to be the most accurate staging technique for neoplasia and is therefore preferred over surveillance biopsies ^79^.

### Endoscopic eradication therapy

More recently, both of the above techniques have been combined to form endoscopic eradication therapy (EET), which has become the standard of care for patients with both LGD and HGD as recommended by the BSG and ACG ^8, 15^. EET consists of endoscopic resection followed by endoscopic ablation. In patients with HGD, a recent systematic review found that EMR followed by RFA resulted in a 93% neoplasia eradication rate and a 73% dysplasia eradication rate ^80^. EET has been associated with a low morbidity and mortality ^81, 82^.

A UK-based cost effectiveness analysis of EET was recently performed. For patients with LGD and HGD, EET was more cost effective than regular endoscopic surveillance and is estimated to provide a 5-year financial benefit to the NHS of £7.1 million ^83^.

## Conclusions

Oesophageal cancer commonly presents late, often after disease has spread and curative treatment is no longer possible. A key issue is identifying at-risk individuals, particularly those with BO, and recent advances in screening methods, including non-invasive strategies, could aid the identification and potential detection of cancer at an earlier stage. This could have a significant impact on survival rates, as emerging early endoscopic interventions have been shown to be successful at eradicating dysplasia and metaplasia. Individuals identified with BO have also been shown to benefit from surveillance strategies, which aim to identify progression from metaplasia to dysplasia. The current universal surveillance strategy with the use of an OGD is time consuming and expensive, but the development of various image enhancement techniques has been proposed to improve its accuracy, reliability, and diagnostic capability.
